# Automatic segmentation of ameloblastoma on ct images using deep learning with limited data

**DOI:** 10.1186/s12903-023-03587-7

**Published:** 2024-01-09

**Authors:** Liang Xu, Kaixi Qiu, Kaiwang Li, Ge Ying, Xiaohong Huang, Xiaofeng Zhu

**Affiliations:** 1 The First Affiliated Hospital of Fujian Medical University, Fuzhou, China; 2 Department of Stomatology, National Regional Medical Center, Binhai Campus of the First Affiliated Hospital, Fujian Medical University, Fuzhou, China; 3 Fuzhou First General Hospital, , Fuzhou, China; 4 School of Aeronautics and Astronautics, Tsinghua University, Beijing, China; 5 Jianning County General Hospital, , Fuzhou, China

**Keywords:** Segmentation, Ameloblastoma, Deep learning, Computed tomography

## Abstract

**Background:**

Ameloblastoma, a common benign tumor found in the jaw bone, necessitates accurate localization and segmentation for effective diagnosis and treatment. However, the traditional manual segmentation method is plagued with inefficiencies and drawbacks. Hence, the implementation of an AI-based automatic segmentation approach is crucial to enhance clinical diagnosis and treatment procedures.

**Methods:**

We collected CT images from 79 patients diagnosed with ameloblastoma and employed a deep learning neural network model for training and testing purposes. Specifically, we utilized the Mask R-CNN neural network structure and implemented image preprocessing and enhancement techniques. During the testing phase, cross-validation methods were employed for evaluation, and the experimental results were verified using an external validation set. Finally, we obtained an additional dataset comprising 200 CT images of ameloblastoma from a different dental center to evaluate the model's generalization performance.

**Results:**

During extensive testing and evaluation, our model successfully demonstrated the capability to automatically segment ameloblastoma. The DICE index achieved an impressive value of 0.874. Moreover, when the IoU threshold ranged from 0.5 to 0.95, the model's AP was 0.741. For a specific IoU threshold of 0.5, the model achieved an AP of 0.914, and for another IoU threshold of 0.75, the AP was 0.826. Our validation using external data confirms the model's strong generalization performance.

**Conclusion:**

In this study, we successfully applied a neural network model based on deep learning that effectively performs automatic segmentation of ameloblastoma. The proposed method offers notable advantages in terms of efficiency, accuracy, and speed, rendering it a promising tool for clinical diagnosis and treatment.

## Background

Ameloblastoma is a benign odontogenic tumor that originates from epithelial cells, specifically from structures such as the lining of enamel organs, dental lamina remnants, and odontogenic cysts. This slow-growing tumor gradually destroys cortical bone and tooth roots, often presenting with symptoms such as facial asymmetry, loose teeth, malocclusion, paresthesia, and pain [1]. It is the second most common odontogenic tumor, accounting for approximately 3%-14% of all jaw tumors and cysts [2]. The annual incidence of ameloblastoma is estimated to be one in every two million individuals [3]. Despite being classified as benign by the World Health Organization, ameloblastoma poses a concern for clinicians due to its locally aggressive nature and the potential for recurrence [4]. Previous studies indicate that about 70% of cases may undergo malignant transformation, with a 2% chance of metastasis [5, 6]. Consequently, radical resection is typically required for ameloblastoma, in contrast to other benign odontogenic tumors. In cases where the lesion size reaches a certain threshold, plate reconstruction or reconstructive surgery may be necessary. According to the meta-analysis authored by Hendra, radical resection was beneficial for reducing the risk of recurrence regardless of the type of ameloblastoma [7]. Hence, achieving precise segmentation of ameloblastoma prior to surgery holds significant clinical implications. Accurate delineation of the tumor's boundaries from medical images is indispensable, as it furnishes clinicians with precise details concerning the tumor's location, dimensions, and morphology. Such information assumes a pivotal role in facilitating accurate diagnosis and differential diagnosis. Furthermore, this segmentation process yields valuable insights that can contribute to optimizing radiation therapy and surgical interventions, thereby enhancing treatment planning efficacy.

Radiology plays a crucial role as a complementary tool to clinical examination, particularly in cases where bone lesions associated with ameloblastoma are challenging to detect during the early stages solely through physical examination [8]. This is particularly relevant for extensive lesions or those accompanied by inflammation, as biopsy results can only provide a reference point. In such cases, radiological examination plays a crucial role in the diagnosis process. However, this step presents certain challenges. Firstly, the radiographic features of ameloblastoma, odontogenic keratocyst, and odontogenic cyst are similar, often making their distinction difficult [9]. Consequently, misdiagnosis is not uncommon in clinical practice. Secondly, early detection of the disease poses challenges, resulting in delays in diagnosis [10]. Finally, the expertise of the imaging physician and the quality of the scanner can influence the diagnostic outcome [11]. Manual interpretation of images is time-consuming, and professional radiologists require extensive training. All these factors contribute to the increased demand for accurate diagnosis of ameloblastoma based on medical imaging. Rapid and accurate methods for diagnosing ameloblastoma using medical imaging hold significant clinical importance.

Since the concept of artificial intelligence (AI) was proposed at the Dartmouth Conference in 1956, AI has made significant progress and is gradually changing every field of human society. In the realm of cysts and tumors of the jaw, AI has shown great potential [12]. In the field of medical imaging, AI techniques can extract vast amounts of information from images such as computed tomography (CT), magnetic resonance imaging (MRI), and panoramic radiographs (PR), enabling lesion segmentation, feature extraction, and disease classification. By digging deep into extensive image datasets, AI can assist doctors in making more accurate diagnoses, predictions, and treatment decisions [13, 14]. In a study conducted by Poedjiastoeti in 2018, VGG-16 architecture was utilized for the differential diagnosis of ameloblastoma and odontogenic keratocyst on PR [15]. The results demonstrated a sensitivity of 81.8%, specificity of 83.3%, accuracy of 83.0%, and a diagnostic time of 38 s. The diagnostic efficiency achieved through AI techniques is comparable to that of oral and maxillofacial surgery, while significantly reducing the diagnostic time. Zijia Liu combined two types of convolutional neural networks (CNN), VGG-19 and ResNet, to construct an AI model [16]. By cropping a 256 × 256 area around the tumor on PR and differentiating between the two tumors, they achieved an improved accuracy of 90.36%. PR has shown positive diagnostic value as a primary screening tool, although it may not be the optimal choice for jaw lesions. On the other hand, CT imaging provides a high-resolution, three-dimensional representation of jaw cysts and tumors without distortion or overlap [17]. Similar studies have been conducted on CT/CBCT images. The results of Bispo's study indicate that Inception v3 has a high classification accuracy for odontogenic keratocysts and ameloblastomas based on CT images [18]. However, the initial dataset of CT images used in the experiment was limited to only 350 images, requiring further expansion. To address this limitation, Chai conducted an improved experiment using Inception v3 [19]. They increased the sample size and employed cropping techniques on cone-beam computed tomography (CBCT) images for differential diagnosis. The results demonstrated higher accuracy, sensitivity, and specificity compared to clinical surgeons. However, these articles primarily focused on the differential diagnosis of ameloblastoma and odontogenic keratocyst, without exploring the accuracy of AI in tumor segmentation. Yet, segmentation serves as the fundamental basis for classification. To the best of our knowledge, there are no existing studies on automatic ameloblastoma segmentation based on CT/CBCT images. Moreover, previous studies primarily utilized horizontal images, lacking coronal and sagittal images.

This paper aims to investigate and evaluate the application of artificial intelligence technology for the automated segmentation of ameloblastoma on CT images. Specifically, we utilize three-dimensional CT images as input for analysis and processing using the Mask R-CNN neural network model. Our goal is to leverage the capabilities of Mask Region-based Convolutional Neural Network (Mask R-CNN) to train a model using a limited dataset, thereby achieving precise and efficient segmentation of ameloblastoma lesions.

## Materials and methods

The study protocol adhered to the principles outlined in the Declaration of Helsinki and received approval from the Ethics Committee of the First Affiliated Hospital of Fujian Medical University (Approval No.IEC-FOM-013–2.0). Since this study was conducted retrospectively, the ethics committee of the First Affiliated Hospital of Fujian Medical University waived the need for informed consent from the participants.

### Data collection

The retrospective analysis involved patients diagnosed with ameloblastoma at the Stomatology Center of the First Affiliated Hospital of Fujian Medical University between 2013 and 2022. Histopathological confirmation of all cases was performed by a pathologist with over ten years of experience. CT data were obtained using an Aquilion TSX-101A device (Toshiba Co., Ltd, Tokyo, Japan) at the Radiology Department. The imaging parameters were set as follows: 120 kV, 40 mA, pulsed scan time of 3 s, 32 × 32 cm2 field of view, and a voxel size of 0.6 mm. Based on inclusion and exclusion criteria, a total of 79 patients were included in the study. The mean age of the subjects was 35.8 years (range from 13–75 years), and there were 50 males and 29 females. Detailed demographic data of the study participants are presented in Table 1. Preoperative CT images of these patients were retrospectively selected, resulting in a total of 3566 images in different orientations (sagittal, horizontal, and coronal), all stored in Digital Imaging and Communications in Medicine format.

**Table 1:** Demographic data of the participants

	n	Proportion (%)
**Gender**		
Male	50	63.29
Female	29	36.71
**Age**		
0–19	20	25.32
20–39	25	31.65
40–59	25	31.65
≥ 60	9	11.38
**Location**		
Maxillary	11	13.92
Mandibular	68	86.08

### Image processing and augmentation

**Fig. 1:**
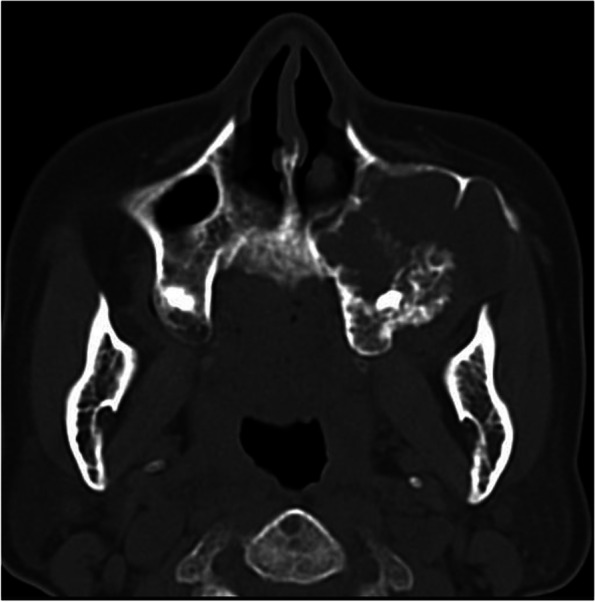
Tumor-centered image cropping with snipraste: A 512 × 512 Dimensional Approach

**Fig. 2:**
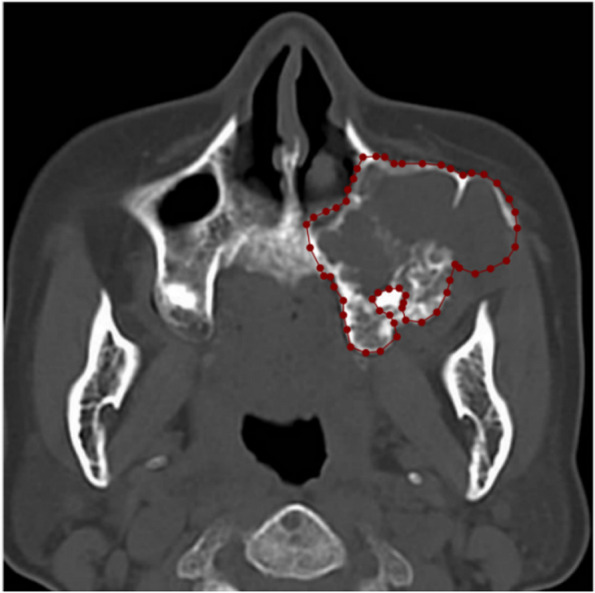
Manual delineation of Ameloblastoma lesions using Labelme Software

Prior to applying deep learning techniques, we performed preprocessing on the included images. The original images were accessed using the Picture Archiving and Communication System developed by YLZ Information Technology company, enabling us to view the images in different orientations. Subsequently, each image was cropped to a tumor-centered image with dimensions of 512 by 512 using the snipraste software, overseen by an oral and maxillofacial surgeon (Fig. 1). Imaging characteristics of each tumor, such as cortical marginal and internal radiolucent lesions, should be included. The cropped images were stored in PNG format. To eliminate dimensional variations among the data, normalization processing was carried out to ensure comparability across different indicators. Linear normalization was implemented in the Aihis software, involving linear transformation of the original data to map the result values between the range of 0 to 1. The normalized images were then imported into Labelme software, where an oral and maxillofacial surgeon with five years of experience manually delineated the edges of the ameloblastoma lesions (Fig. 2). These annotations were reviewed and adjusted by a senior surgeon. The labeled data were randomly divided into a training set (2854 images), a validation set (356 images), and a test set (356 images) in an 8:1:1 ratio. During the training process, data augmentation techniques were applied to reduce redundancy and prevent model overfitting. The data augmentation methods employed in our study included resizing, random flipping, and padding. As a result, the dataset was augmented by 10 times, with a total of 3566 images ultimately utilized for constructing the model.

### Mask R-CNN Architecture and Workflow

**Fig. 3:**
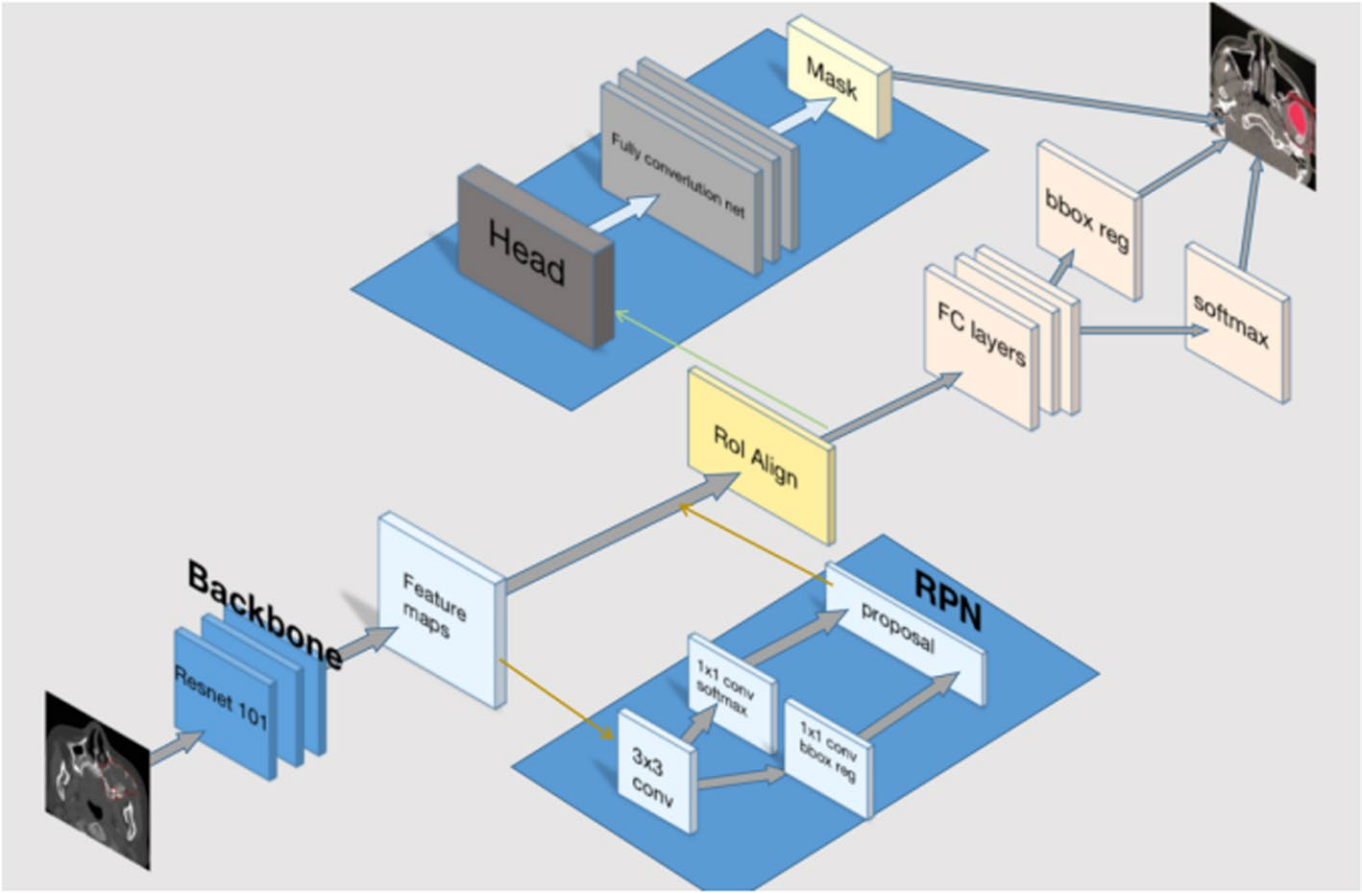
The workflow of Mask R-CNN

Mask R-CNN is an advanced technique for instance segmentation that combines region proposal and convolutional neural network. It allows marking the specific category of the target on each pixel, enabling tasks such as category detection, image segmentation, and feature point positioning. In our study, the pre-processed images were fed into the Mask R-CNN network for transfer learning. The workflow of Mask R-CNN is illustrated in Fig. 3. The main components of Mask R-CNN include the backbone network, regional proposal network (RPN), and head. For our article, the backbone network consisted of a 101-layer deep residual network (ResNet101) combined with a feature pyramid network (FPN).

The ResNet101 backbone network was utilized to extract features from different layers of CT images of ameloblastoma. These features captured important semantic and spatial information about the lesions. FPN then combined the features from different layers to create a feature map that contained crucial information at multiple scales. RPN played a key role in identifying potential regions of interest (ROI) within the image. It employed a sliding window technique to scan the image and determine areas where targets, in this case, ameloblastoma, might be present. The features extracted by the backbone network were fed into the RPN block, which performed two primary tasks: classification and bounding box regression. During the classification process, each anchor (a predefined bounding box) was evaluated to determine whether it represented a background or a foreground (potential ameloblastoma) region. The RPN assigned a classification score to each anchor based on its likelihood of containing the target. Simultaneously, the RPN also estimated the bounding box coordinates around the object within each anchor. These coordinates defined the spatial extent of the potential ROI. By calculating the intersection ratio between each anchor box and the ground truth on the image, multiple candidate ROIs of varying sizes were obtained. Overall, the combination of ResNet101, FPN, and RPN allowed for the extraction of informative features, identification of potential ameloblastoma regions, and localization of precise bounding boxes around these regions for further analysis and segmentation.

In the final step of our methodology, the region proposals obtained from the RPN were further processed and analyzed in the head block of the Mask R-CNN model. Approximately 2000 region proposals were extracted from each image by filtering out regions with low scores. Each region proposal was then warped to a fixed size of 227 × 227 pixels and input into the head block. To align the ROI for accurate feature extraction, we employed ROIAlign, a technique that transformed regions of different sizes on the feature map into a uniform size. By utilizing bilinear interpolation, each pixel within the ROI was calculated based on adjacent grid points on the feature map, allowing us to capture critical feature information specific to the region of interest. In this step, we performed tumor classification, regression, and segmentation tasks. By adding a segmentation branch on the fully connected layer, we classified and predicted each pixel within the ROI, generating a binary mask as the final output. This mask delineated the boundary of the ameloblastoma region. To optimize the model's performance, we conducted training and validation iterations for 100 epochs, using augmented data. The learning rate of the model was set at 0.005 (SGD). The weight decay was set at 0.0001. To evaluate the effectiveness of the trained model, we performed ten-fold cross-validation. This rigorous training and evaluation process ensured that the model was optimized to accurately classify and segment ameloblastoma lesions in CT images, providing reliable results for clinical diagnosis and treatment planning. Throughout the training process, the validation loss steadily decreased, indicating improved accuracy and convergence. To evaluate the model's generalization performance, we acquired an additional dataset of 200 CT images of ameloblastoma from a different dental center. We followed the same experimental procedures as before, repeating the steps to assess the model's performance on this new dataset. This validation process allowed us to examine how well the model could handle data from an external source and provided insights into its robustness and applicability across different datasets. Our model was trained under the Ubuntu operating system, version 18.04, with the graphics card being the NVIDIA TITAN RTX 24G.

### Statistical analysis

The performance evaluation of Mask R-CNN in this study involved the utilization of Dice coefficient and average precision. The Dice coefficient serves as a commonly employed metric in medical image segmentation, functioning as a measure of set similarity between two samples within a threshold range of [0,1]. Its primary application lies in medical image segmentation, where a Dice coefficient of 1 represents optimal segmentation performance, while a value of 0 indicates the poorest result. The Dice coefficient is mathematically defined as follows:

$$\mathrm{Dice}\left(\mathrm X,\mathrm Y\right)=\frac{2\mathrm{IX}\cap\mathrm{YI}}{\mathrm{IXI}+\mathrm{IYI}}$$

The Average Precision (AP) is a performance metric commonly used to assess the effectiveness of object detection or image segmentation models. In tasks involving object detection or image segmentation, the model generates a set of bounding boxes or pixel masks, which need to be compared with the ground truth targets. The IoU (Intersection over Union) is computed between the model's prediction and the actual target, and the prediction is considered accurate if the IoU exceeds a predefined threshold. To evaluate the model's performance at various thresholds, we calculate different combinations of recall and precision. Plotting these values results in a Precision-Recall (PR) curve, and the area under this curve corresponds to the Average Precision.

## Result

**Fig. 4:**
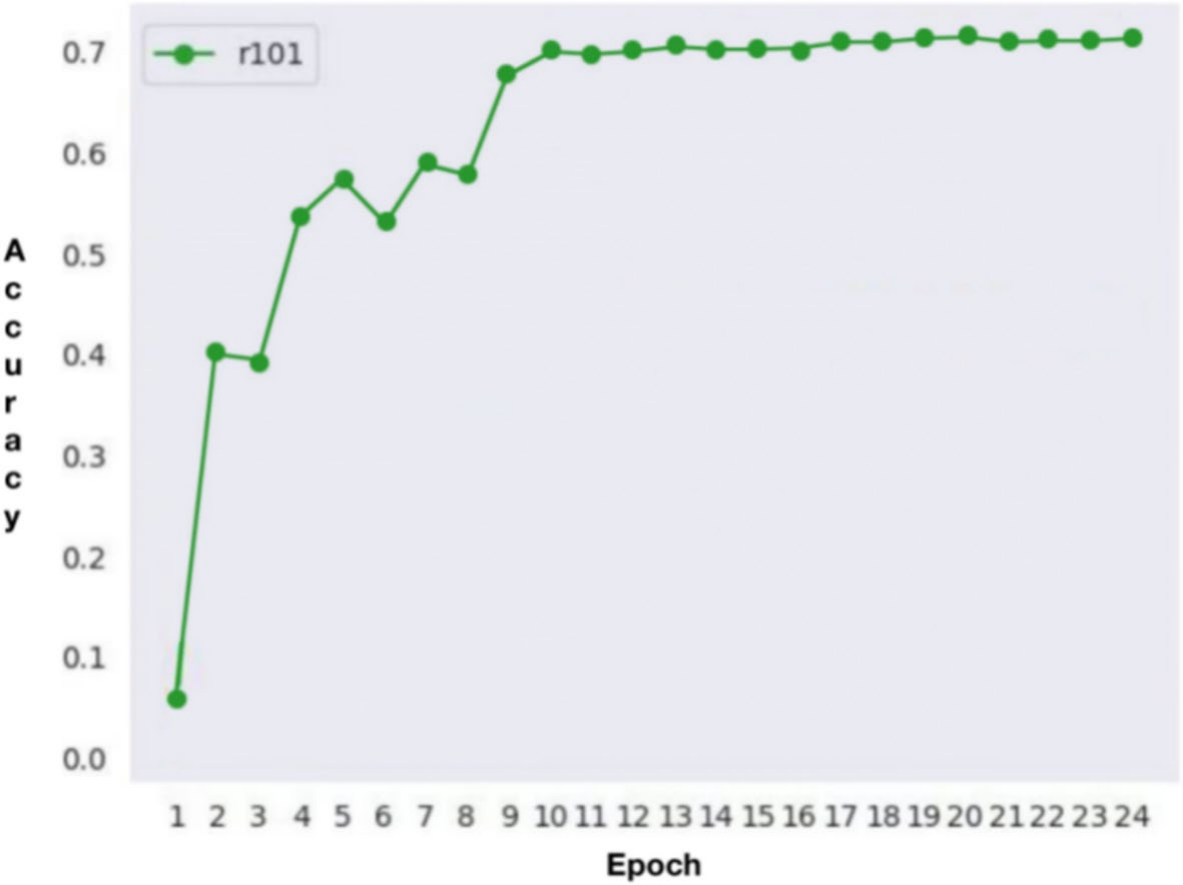
Accuracy VS epochs

**Fig. 5:**
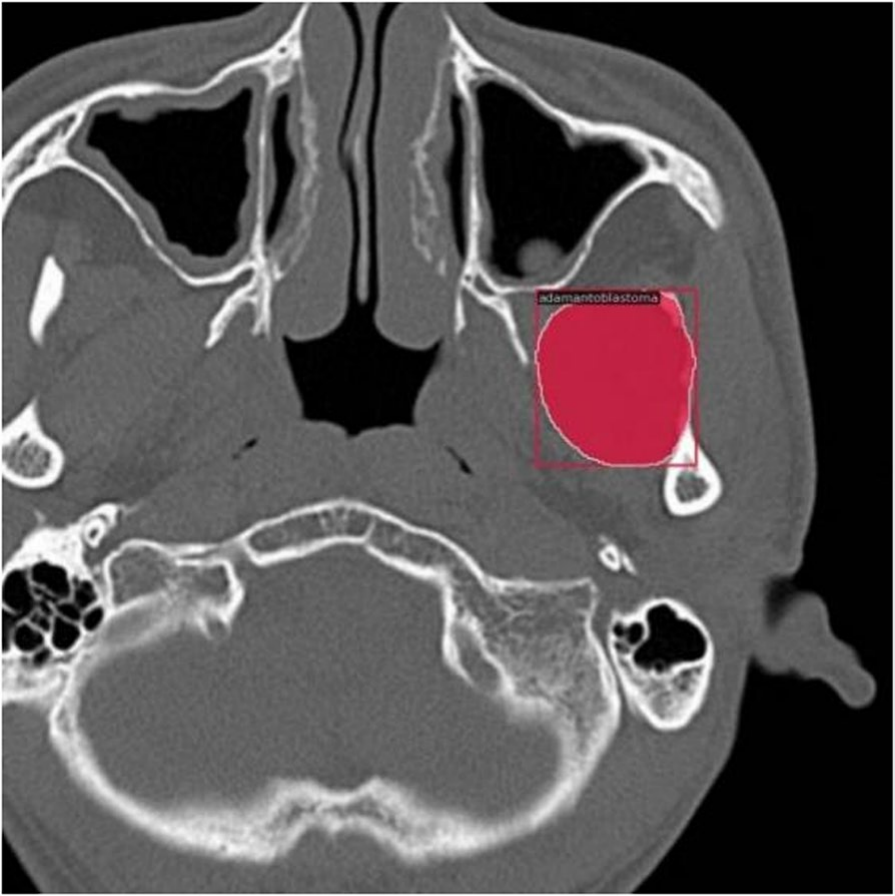
Ameloblastoma was identified and segmented by Mask R-CNN model

**Fig. 6:**
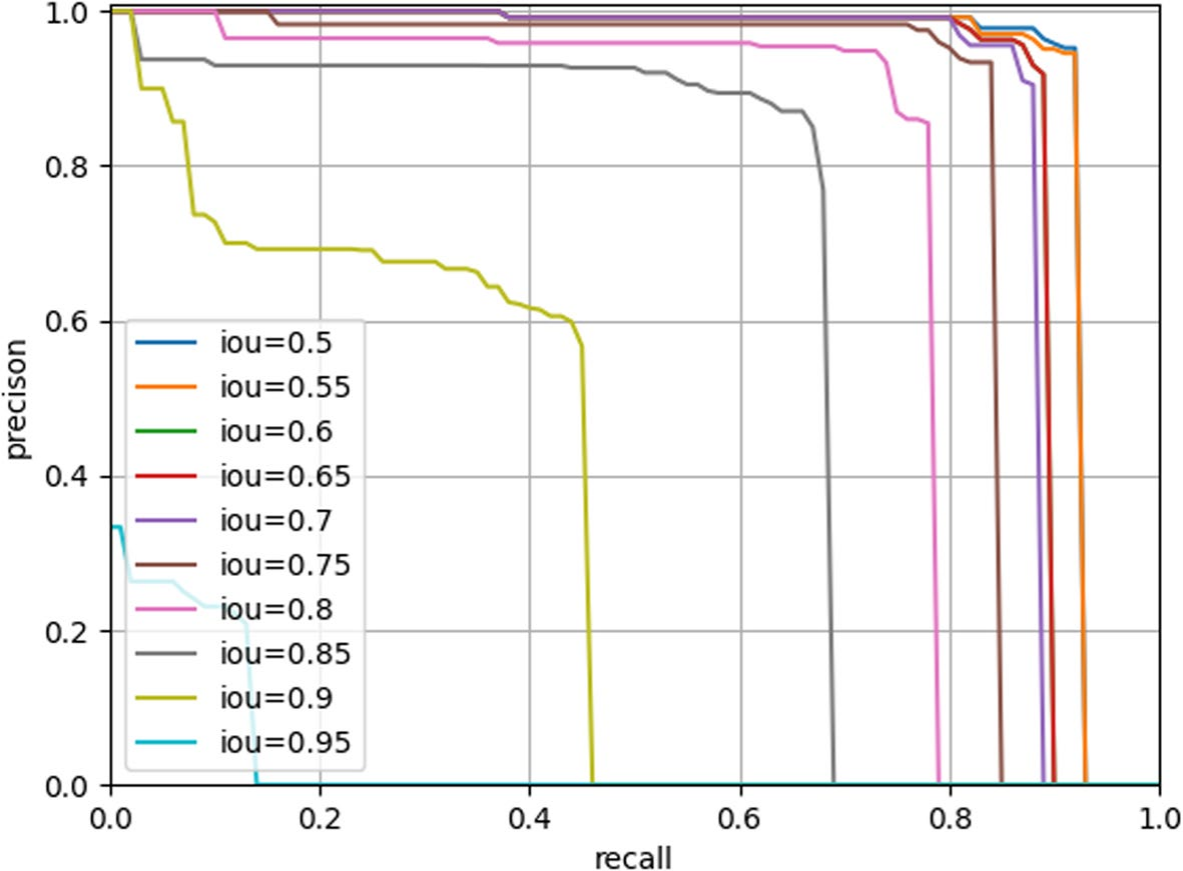
Precision-Recall curves of model for different IoU

**Fig. 7:**
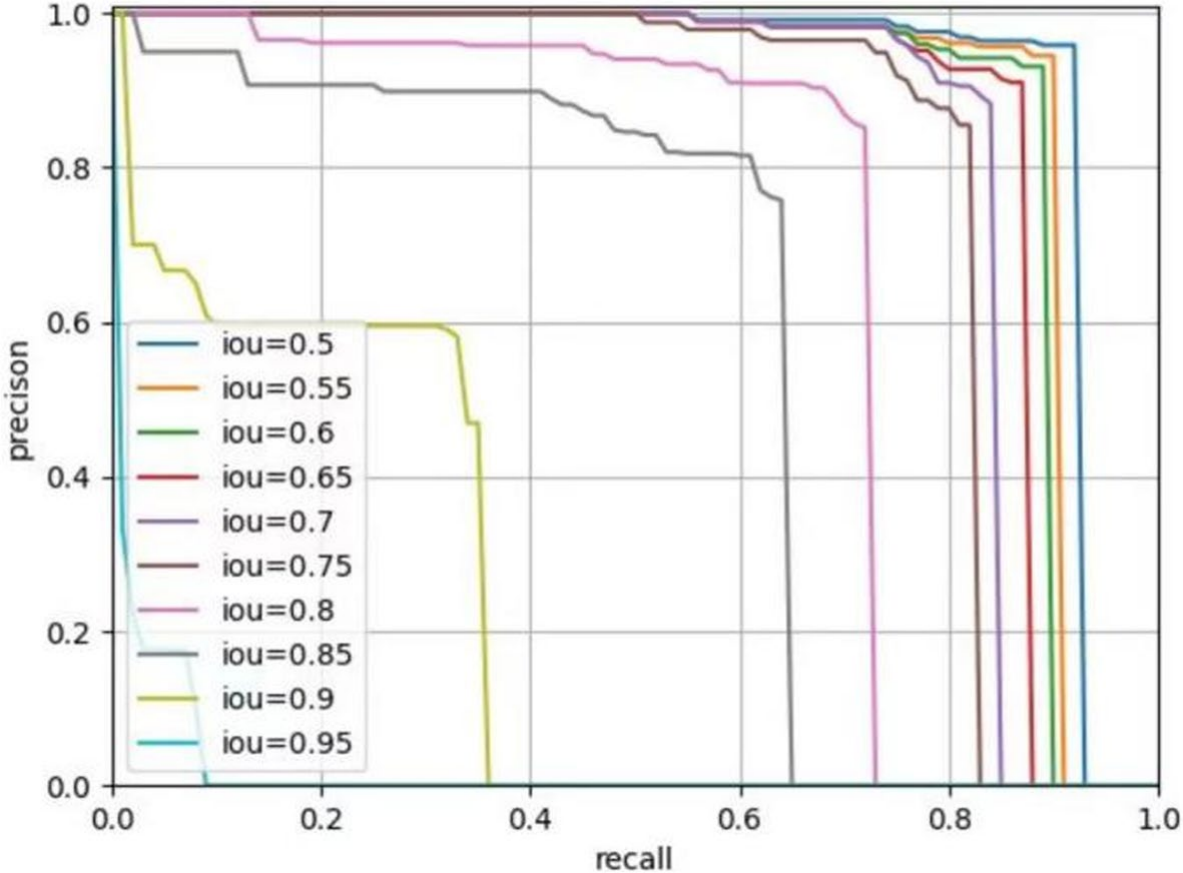
The generalization performance of the Mask R-CNN model

In the process of building the model, we initially set the training to run for 100 epochs. Surprisingly, the accuracy had already reached a satisfactory level by the 20th epoch (Fig. 4). This outcome underscores the effectiveness of our chosen loss function configuration, which facilitated rapid model convergence and achieved excellent accuracy within a concise training period. During the segmentation process, the model showcased remarkable efficiency, taking a mere 0.1 s to segment an image. This swift performance significantly reduces the operational time compared to manual segmentation methods, thereby endowing it with substantial practical advantages. Figure 5 shows the segmentation of ameloblastoma on CT images by Mask R-CNN model. After extensive testing and evaluation, our model exhibited remarkable proficiency in automatically segmenting ameloblastoma. The DICE index achieved an impressive value of 0.874. Moreover, when the IoU threshold ranged from 0.5 to 0.95, the model's AP was 0.741. For a specific IoU threshold of 0.5, the model achieved an AP of 0.914, and for another IoU threshold of 0.75, the AP was 0.826 (Table 2). As the IoU threshold changes, the model's PR curve also varies accordingly. From Fig. 6, we can observe that when the IoU is set between 0.5 and 0.75, the precision remains consistently high while the recall value increases. After the recall exceeds 0.7, the precision gradually decreases, and once the recall surpasses 0.8, the precision decreases significantly. Even when the IoU is set to 0.85, the resulting PR curve remains satisfactory. These results indicate the model's ability to attain a high level of precision and accuracy in object detection or image segmentation tasks across various IoU thresholds. However, as the IoU exceeds 0.9, the model's performance becomes increasingly unstable, and the area under the PR curve exhibits a steep reduction. In the evaluation of the model's generalization performance, we generated similar PR curves (Fig. 7). Comparing with Fig. 6, we observed that when the IoU falls within the range of 0.5 to 0.75, all curves exhibit consistent patterns, and their corresponding AP values are quite close. However, when the IoU reaches 0.9, the corresponding PR curve becomes highly irregular.

**Table 2:** Performance metrics for automatic segmentation of ameloblastoma

Dice coefficient	AP(IoU = 0.5–0.95)	AP(IoU = 0.5) (IOU = 0.50:0.95)	AP(IoU = 0.75)	time (s)
0.874	0.741	0.914	0.826	0.332

## Discussion

Tumor segmentation plays a crucial role in medical image analysis [20]. However, to the best of our knowledge, there have been no studies focusing on the application of AI in ameloblastoma segmentation, although similar studies have been conducted on other types of tumors. For instance, Abdolali et al. proposed an asymmetric analysis-based method for automatic segmentation of jaw cysts, which demonstrated favorable performance in experimental results [21]. In the case of keratocystic odontogenic tumor, the average Dice coefficient achieved was 0.80. Furthermore, Paderno et al. achieved a Dice coefficient of 0.65 for the AI model in segmenting oral squamous cell carcinoma, as observed in their experiment [22]. Those experimental results provide evidence of the effectiveness of AI in segmenting maxillofacial tumors. It is widely recognized that the clinical application of segmentation becomes more valuable as the efficiency of the process improves. In our research, we proposed a Mask R-CNN model specifically designed for automatic segmentation of ameloblastoma based on CT images. In terms of accuracy assessment, it is generally considered reliable when the intersection over union value exceeds 0.7 [23]. Our experimental results demonstrate that when the IoU is set to 0.75, the model achieves accurate segmentation with an AP value of 0.826. This indicates the effectiveness of Mask R-CNN in accurately partitioning ameloblastoma. This success may be attributed to the utilization of Mask R-CNN, which is a versatile and compact framework for object instance segmentation. This model not only detects targets within the image but also provides pixel-level segmentation results for each individual target [24]. The PR curves clearly indicate that the model's performance starts to decrease significantly when the IoU is set above 0.9. On the other hand, the model consistently demonstrates reliable performance when dealing with IoU values between 0.5 and 0.75. The results suggest that the AI model we applied exhibits a relatively accurate segmentation ability for ameloblastoma, although it may not possess an absolute accuracy in segmentation. Furthermore, we assess the generalization of our model through the evaluation of externally validated datasets, and the results are also satisfactory. This indirectly indicates that the model possesses good robustness.

Previous studies commonly employed traditional semantic segmentation models in similar contexts. However, these methods often face challenges related to imbalanced classification [25]. In contrast, our study utilizes Mask R-CNN, an instance segmentation framework known for its superior feature extraction capability. By selecting Mask R-CNN as our deep learning model, we benefit from its ability to provide accurate bounding boxes and pixel masks for each target, enabling pixel-level segmentation [26]. Furthermore, Mask R-CNN exhibits versatility in handling targets of different sizes, shapes, and quantities, making it highly adaptable across various application scenarios, including medical image segmentation [27]. During our investigation, we found that three scenarios may lead to less efficient segmentation. Firstly, when the tumor is situated in the maxilla, challenges arise due to the thinner bone cortex of the maxilla and the proximity of multiple sinus cavities. Maxillary lesions often lead to the destruction of the nasal cavity, ethmoid sinus, and sphenoid sinus, causing the tumors to locally extend into the sinus cavity. As a result, the model faces difficulty in accurately distinguishing the boundary between the sinus cavity and the tumor. Secondly, when the tumor extensively destroys the jaw cortex, the damaged jaw bone loses its structural continuity, posing difficulties for both human experts and AI algorithms in accurately determining the tumor boundary. Lastly, in cases where a section of the ameloblastoma boundary overlaps with the crown or root of a tooth, the precise distinction becomes challenging due to the high density exhibited by both structures on imaging.

In our study, CT imaging was selected over cone-beam computed tomography (CBCT) for the diagnosis of jaw tumors due to its unique advantages. Firstly, CT offers higher spatial resolution and a wider scanning range [28], enabling more accurate segmentation of jaw tumors and facilitating the development of precise treatment plans for ameloblastoma. Secondly, CT images exhibit a highly linear relationship between pixels, which enhances the stability and reliability of computer analysis and reconstruction [29]. Moreover, unlike previous experiments that utilized only horizontal CT images, our study employed three-dimensional CT images, providing a more comprehensive representation of the anatomical structures and pathology. This allowed our model to capture additional spatial information, further enhancing its performance.

The training of deep learning models typically relies on large-scale datasets to ensure the robustness of the final results [30]. However, certain rare medical conditions, like the ameloblastoma studied in this research, often present limited sample sizes that do not meet the requirements for deep learning. Consequently, there has been a growing research focus on leveraging deep learning techniques with limited sample sizes, resulting in notable advancements in this field in recent years [31, 32]. Nonetheless, deep learning based on small samples faces its own challenges, with overfitting and data imbalance being among the most common issues [33]. To address these concerns during the construction of Mask R-CNN, we fine-tuned the classifier's weights and employed data augmentation techniques to mitigate the impact of data imbalance. Furthermore, we obtained images from other medical centers to assess the model's extrapolation capability, which ultimately yielded satisfactory results.

Several studies have explored the application of artificial intelligence and radiomics in the diagnosis and treatment of ameloblastoma, with a focus on differential diagnosis. Gomes et al. conducted research on MRI texture analysis, including contrast, entropy, and homogeneity, as a tool for distinguishing ameloblastoma from odontogenic keratocyst, achieving a diagnostic efficiency of 83.3% [34]. In another study, researchers utilized the VGG-16 model for jaw tumor classification based on panoramic images, but the diagnostic efficiency and time of the AI model did not exhibit significant advantages compared to manual diagnosis [15]. Subsequently, Liu et al. improved the experiment by comparing four deep learning models and found that a convolutional neural network structure based on transfer learning algorithm could accurately differentiate ameloblastoma from odontogenic keratocyst with an accuracy of 90.4% [16]. Similar studies utilizing CT images have also demonstrated relatively high accuracy levels [18, 19]. However, accurate segmentation is a crucial prerequisite for AI models to effectively classify medical images and produce reliable results. In other fields, the segmentation and classification of tumors by artificial intelligence on medical images have been intensively studied [35, 36]. Therefore, the significance of our study lies in addressing this research gap by applying a segmentation approach for ameloblastoma. By accurately separating ameloblastoma from medical images, our model can assist doctors in precisely determining the tumor's location, size, and shape. This information holds great importance for accurate diagnosis and differential diagnosis. Furthermore, it can provide valuable insights for radiotherapy and surgery, enabling better treatment planning. Additionally, tumor segmentation can contribute to patient prognosis assessment and aid in estimating the probability of recurrence. Manual interpretation in radiology often involves perceptual errors, which account for a substantial portion of misdiagnoses [37]. AI has the potential to effectively address this issue [38]. Our model demonstrates remarkable efficiency in the segmentation process, requiring only 0.1 s per image. This significant reduction in operational time compared to manual segmentation methods makes our approach highly time-effective.

However, this study does have certain limitations that should be acknowledged. The samples used in this experiment were obtained from a single center, which may introduce a bias and limit the extrapolation ability of the model to diverse populations or different imaging protocols. Further research involving multiple centers and a more diverse patient population would be beneficial to explore the model's generalizability and assess its performance in different clinical settings.

## Conclusion

It seems that deep learning holds significant potential in aiding oral general practitioners and junior maxillofacial surgeons in the swift detection and segmentation of ameloblastoma, thereby facilitating early diagnosis and treatment. Furthermore, this study introduces a novel approach and methodology for the application of computer-aided diagnosis in the realm of oral and maxillofacial surgery, with the potential for widespread adoption and implementation.

## Data Availability

The datasets used and/or analyzed during the current study available from the corresponding author on reasonable request.

## Abbreviations

AI: Artificial intelligence
CT: Computed tomography
CBCT: Cone-beam computed tomography
RPN: Regional proposal network
ResNet101: 101-Layer deep residual network
FPN: Feature pyramid network
Mask R-CNN: Mask Region-based Convolutional Neural Network
ROI: Regions of interest
IoU: Intersection over Union
